# Growth‐restricted human fetuses have preserved respiratory sinus arrhythmia but reduced heart rate variability estimates of vagal activity during quiescence

**DOI:** 10.14814/phy2.15458

**Published:** 2022-11-21

**Authors:** Anne Rahbek Zizzo, John Hansen, Olav Bjørn Peteren, Henning Mølgaard, Niels Uldbjerg, Ida Kirkegaard

**Affiliations:** ^1^ Department of Obstetrics and Gynaecology Aarhus University Hospital Aarhus N Denmark; ^2^ Department of Clinical Medicine Aarhus University Aarhus Denmark; ^3^ Department of Health Science and Technology Aalborg University Aalborg Denmark; ^4^ Department of Obstetrics Copenhagen University Hospital Copenhagen Denmark; ^5^ Department of Clinical Medicine University of Copenhagen Copenhagen Denmark; ^6^ Department of Cardiology Aarhus University Hospital Aarhus N Denmark

**Keywords:** cardiac vagal activity, fetal electrocardiography, fetal growth restriction, fetal physiology, respiratory sinus arrythmia, spectral domain, time domain

## Abstract

The aim was to assess the association between fetal growth restriction (FGR) and fetal heart rate variability (FHRV) in relation to fetal movements. A prospective observational cohort study was performed. Non‐invasive fetal electrocardiography (NI‐FECG) allowed beat‐to‐beat assessments with <5% corrections of RR intervals. FHRV analyses included: Root mean square of successive RR interval differences (RMSSD), high frequency power (HF power), and low frequency power (LF power). Fetal movements were categorized by continuous ultrasound scanning. We enrolled 36 singleton pregnant women expecting a small fetus (< the 2.3 percentile of mean weight for gestational age) diagnosed by ultrasound, of whom 25 presented with a birthweight < the 2.3 percentile. Among these, 11 were excluded due to low quality NI‐FECG recordings, leaving 14 women with 28 recordings eligible for inclusion in the analyses. The control group consisted of 22 healthy fetuses with birthweights between the 10th and the 90th percentile (average for gestational age [AGA]). In FGR fetuses the HRV response to respiratory activity was comparable to that of AGA fetuses. RMSSD (Ratio 1.54 [95% CI: 1.33; 1.79]) and HF power (Ratio 2.88 [95% CI: 2.12; 3.91]) increased, whereas LF/HF power (Ratio: 0.44 [95% CI: 0.31;0.63]) decreased. However, during fetal quiescence, FGR fetuses differed significantly from AGA fetuses. Compared to AGA fetuses, FGR fetuses displayed lower RMSSD (Ratio 0.77 (95% CI: 0.58; 1.02)) and HF power (Ratio 0.56 (95% CI:0.32; 0.98)). This reduction was associated with the severity of the FGR. In conclusion, FGR fetuses displayed a respiratory sinus arrhythmia (RSA) comparable to AGA fetuses; however, more important, parameters representing cardiac vagal activity were impaired in FGR fetuses during quiescence. RSA may constitute an intrinsic function of the cardiovascular system, which is unaffected by fetal compromise. However, the basic cardiac outflow assessed during fetal quiescence indicates a suppressed cardiac vagal activity in the FGR fetuses.

## Graphical abstract

Growth restricted human fetuses displayed a respiratory sinus arrhythmia (RSA) comparable to average for gestational age fetuses; however, more important, HF power and RMSSD were impaired in FGR fetuses during quiescence, indicating a suppressed cardiac vagal activity.

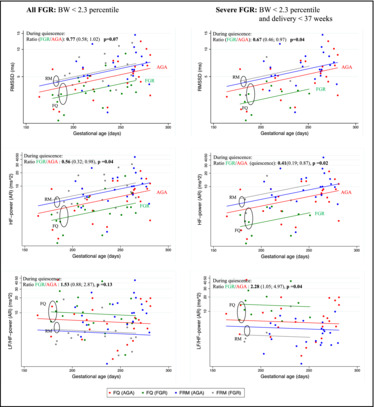

## 1 INTRODUCTION

Despite advances in fetal monitoring, fetal growth restriction (FGR) is still a leading cause of perinatal death, neonatal complications, and long‐term neurological disabilities (Baschat, 2011; von Beckerath et al., 2013). As intrauterine treatment is less established (Hansen et al., 2018), fetal surveillance and optimal timing of delivery without neurological damage is crucial to neonatal outcome and has been addressed by several studies (Donchin et al., 1984; GRIT Study Group, 2003; Lees et al., 2015). However, optimal timing of delivery remains a great challenge.

Fetal defense against hypoxia includes a carotid chemoreflex triggered redistribution of blood flow away from peripheral vascular beds and toward vital organs including the brain (Giussani, 2016). This phenomenon called “brain sparring” and the cardiac failure following hypoxia are clinically revealed by Doppler flow assessments of fetal vessels. Among these, late ductus venosus (DV) flow changes have been shown to be a promising predictor of major neonatal complications such as death and respiratory distress syndrome (RDS) (Bilardo et al., 2004). However, the fetal neurological state is less revealed by Doppler flow measurements.

Conventional cardiotocography (CTG) displays the “baseline variability” of fetal heart rate, which for decades has been used to assess fetal neurological wellbeing, and especially the risk fetal hypoxia (Hon & Lee, 1963). Nevertheless, this method has been shown to be less predictive of neonatal outcome (Alfirevic et al., 2017), which to some extent may be due to high interobserver variation (Rhose et al., 2014). Computerized CTG (cCTG) was developed to bring objectivity into CTG interpretation. Based on big data, thresholds of low short‐term variability (STV), depending on gestational age, have been developed, and a randomized controlled trial (TUFFLE) concluded that combining late DV changes and low STV improved 2‐year neurodevelopmental outcomes (Lees et al., 2015). However, post‐hoc analyses revealed that STV alone was unable to predict hypoxia, acidosis, and neurodevelopment (Wolf et al., 2017).

STV by cCTG is based on an averaging of fetal heart rate, but it does not provide a beat‐to‐beat evaluation of fetal heart rate variability, which in adults has been shown to be of significant importance (Task Force oTESoCatNASfPaE, 1996). The emerging interest in beat‐to‐beat FHRV based on non‐invasive electrocardiography (NI‐FECG), which is obtained by electrodes placed on the maternal abdomen, has revealed its potential as a clinical diagnostic measure as the method is feasible and the results are reliable (Rahbek Zizzo et al., 2022; Zizzo et al., 2022). Recent studies of healthy fetuses have shown that by standardizing fetal respiratory movements, reliability increases and a respiratory‐dependent sinus arrythmia (RSA) is revealed. (Rahbek Zizzo et al., 2022; Zizzo et al., 2020). In adults, RSA is closely linked to health and prognosis in adverse diseases (Cygankiewicz & Zareba, 2013).

The aim of the present study was to assess the association between FHRV and FGR in relation to fetal movements and further, to evaluate the diagnostic accuracy of FHRV in predicting FGR.

## 2 METHODS

### 2.1 Participants

In this prospective observational cohort study, we included 36 singleton pregnant women with an estimated fetal weight (EFW) below the 2.3 percentile of mean weight for gestational age (−22%) and a gestational age (GA) > 23^+6^ weeks. EFW was computed by biparietal diameter, head circumference, abdominal circumference, and femur length (Hadlock et al., 1991). All women had attended the national screening program at Aarhus University Hospital, which included a combined screening test for chromosomal abnormalities, based on maternal age, the serum PAPP‐A and free β‐hCG measured between GA 8^+0^ and 13^+6^, and the nuchal translucency assessed by ultrasound in GA 11^+3^–13^+6^. In gestational weeks 19–20 an additional ultrasound scan, containing a screening for malformations, was performed. In case of high‐risk of chromosomal abnormalities, array comparative genomic hybridization (Array‐CGH) was offered. The estimated due date was determined by means of the crown rump length (CRL) using the formula of Robinson et al. at the first trimester scanning (Robinson & Fleming, 1975).

Exclusion criteria were insufficient quality of the NI‐FECG recordings (defined below), birth weight above the 2.3 percentile corrected for gestational age and gender (Marsál et al., 1996), fetal malformations, and chromosomal abnormalities diagnosed during pregnancy or within the first neonatal week. To evaluate FHRV in relation to the severity of FGR, one subgroup was defined: fetuses born < GA 37^+0^ and with a BW < the 2.3 percentile (in this study termed severe FGR). These criteria were based on the assumption that a preterm delivery reflects the total obstetric assessment of a compromised fetus, as preterm delivery in FGR is mostly induced by indication.

A control group consisted of 22 healthy singleton pregnant women carrying an appropriate for gestational age (AGA) fetus defined by a birthweight between the 10th and 90th percentile of mean for gestational age and gender, delivered at term (GA 37^+0^–41^+0^) and with no fetal malformations or chromosomal abnormalities. These women were included between GA 24^+0^ and 40^+1^. Data on the AGA fetuses have been submitted in a study assessing the reliability of FHRV in heathy fetuses (Rahbek Zizzo et al., 2022).

For all included women, caffeine intake, smoking, and high intensity exercise were registered at every assessment by hours since last exposure.

### 2.2 Acquisition of NI‐FECG and fetal movements

In women expecting a small fetus, NI‐FECG recordings were obtained just before or after the scheduled ultrasound assessments in the ultrasound clinic. All women were assessed with a maximum interval of 14 days, but the interval between the recordings varied between the included women, as did the number of recordings (range 1–5 recordings). A small recorder (Viewcare A/S, Søborg, Denmark) obtained the NI‐FECG by electrodes placed on the maternal abdomen and with a sampling frequency of 1 kHz and a resolution of 24‐bit. All recordings were of 20 minutes duration and performed during the daytime (8 a.m. to 4 p.m.). Filters consisted of a notch filter (50 Hz), a low‐pass filter (5 Hz) and a high‐pass filter (150 Hz). The algorithm for fetal R‐wave detection was developed by Viewcare (Viewcare A/S, Søborg, Denmark), based on templates of fetal and maternal QRS‐complexes (Sæderup et al., 2019).

Fetal movements were detected by continuous ultrasound scanning (Voluson E10, GE Healthcare) performed simultaneously with the NI‐FECG recording. A sagittal view of the fetus, including fetal diaphragm movements, was displayed. Fetal gross movements (extremities and trunk) were also visible in this scanning position. At every 15‐second time period, fetal movements were divided into one of three categories: (1) fetal body movements (FBM), defined by continuous fetal movements of the trunk and/or extremities, (2) fetal respiratory movements (FRM), defined by continuous movements of the thoracic diaphragm and no other body movements, or (3) fetal quiescence (FQ), defined by no fetal body or respiratory movements except for occasional kicks or startles.

The procedures for obtaining NI‐EFCG recordings, detecting fetal movements, and dividing fetal movements into three categories were the same in AGA and FGR fetuses. (Rahbek Zizzo et al., 2022)

### 2.3 Processing and selection of time series

Analyses of FHRV were performed using the Kubios Premium software (Kubios heart rate variability software version 3.3; Biosignal Analysis and Medical Imaging Group, Department of Physics, University of Kuopio, Kuopio, Finland). The signal was detrended by the smoothen priors regulation (Tarvainen et al., 2002). Cubic spline interpolation was used for artifact correction (missing beats, ectopic beats or extrasystoles, Daskalov & Christov, 1997; Mateo & Laguna, 2000). In most recordings a threshold of 40 milliseconds for RR‐wave correction was appropriate. This means that an RR interval was corrected if it deviated more than 40 milliseconds from the preceding RR interval. However, in a few recordings, a threshold of 100 milliseconds was necessary to ensure that only artifacts were corrected. R‐wave corrections were visually confirmed in all included time series. In the spectral analyses, RR intervals were re‐sampled at 4 Hz.

**FIGURE 1:**
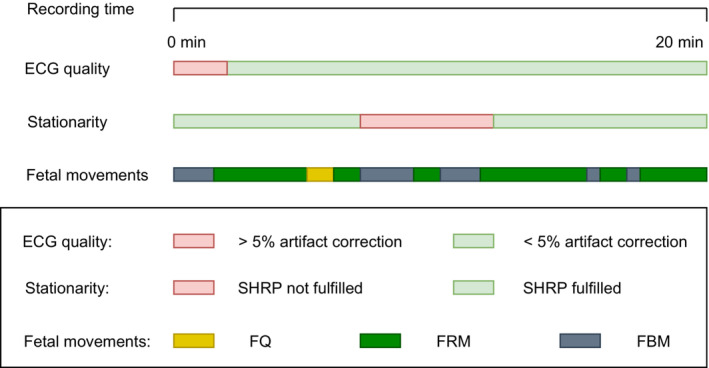
Illustration of the criteria used for selection of included time series, in relation to recording time. Legends: ECG: Electrocardiography, SHRP: Stationary heart rate pattern defined as no accelerations or decelerations and floating of baseline less than 10 bpm/2 min, Fetal movements: Three categories, based on ultrasound: FQ, fetal quiescence; FRM, fetal respiratory movements; FBM, fetal body movements.

Included time series fulfilled the following criteria: (1) <5% correction of RR intervals, (2) stationary heart rate pattern (SHRP), evaluated on a CTG‐like pattern and defined by no accelerations or decelerations (±15 beats per minute [bpm]/15 s) and a stable baseline (floating less than 10 bpm/2 min; Zizzo et al., 2022), and (3) one of the three categories of fetal movements (Figure 1).

We used 64‐s time series, which was found to be sufficient in our former studies regarding both reliability and interpretation (Rahbek Zizzo et al., 2022).

Time domain analyses were mean RR interval (mean RR [ms]), standard deviation of normal to normal RR intervals (SDNN [ms]), and the root mean square of successive RR interval differences (RMSSD [ms]). Spectral domain analyses were high frequency power (HF power [ms^2^]), low frequency power (LF power [ms^2^], and the ratio LF/HF power). The autoregressive model (AR) was used (order set to 24; Task Force oTESoCatNASfPaE, 1996).

The frequency‐bands were set, based on the frequency of fetal respiratory movements (Dornan et al., 1984) and according to former studies (Groome et al., 1994; Gustafson et al., 2011; Rahbek Zizzo et al., 2022; Zizzo et al., 2020): LF power: 0.04–0.4 Hz, HF power: 0.4–1.5 Hz.

RMSSD and HF power are generally accepted as vagally mediated parameters closely linked to RSA, whereas SDNN and LF power probably reflect both sympathetic and parasympathetic activity (Task Force oTESoCatNASfPaE, 1996).

### 2.4 Statistics

All variables were normally distributed when transformed to the natural logarithm. A linear correlation between all parameters and gestational age was found. However, for each parameter no correlation between fetal movements (FRM or FQ) and gestational age was found; thus, identical development (equal slopes) throughout gestational age was accepted for FRM and FQ in each parameter in both AGA and FGR fetuses.

The multivariate mixed model was used due to the presence of correlation within and between fetuses and recording day. Fetal respiratory movements (FRM) were compared to fetal quiescence (FQ) for each parameter in the FGR fetuses, and the differences (back transformed to ratios) were estimated with 95% CI. Furthermore, FGR fetuses were compared to AGA fetuses, both during FQ and FRM, and the differences (back transformed to ratios) were estimated with 95% CI. A *p*‐value <0.05 was interpreted as significant.

For receiver operating characteristics (ROC curves), predicted FGR was obtained using linear regression according to GA. The standardized difference between the observed parameter and the predicted value was used as a predictor for severe FGR (preterm and BW < −22%). ROC curves were plotted to assess the sensitivity and specificity as the function of thresholds of the predicter. The overall prediction ability of the predictor was assessed using the area under the ROC curve (AUC). The unit of analyses was each recording of the fetus. We applied non‐parametric bootstrap to adjust AUC confidence intervals for clustering (correlation) of fetuses.

## 3 RESULTS

We included 14 women with a total of 28 recordings in the analyses. Among the 36 women accepting participation in the study, 11 were excluded due to a birth weight above ‐ 22%, and 11 were excluded due to technicalities: 4 women because no fetal R‐waves could be detected, and 7 women because more than 5% of fetal R‐waves needed correction. The mean number of recordings per woman included in the analyses was 2.0 (Range: 1–5). Gestational age at recording ranged from 26^+1^ to 40^+0^. Characteristics of the included women and the control group (AGA) are shown in Table 1.

**TABLE 1:** Characteristics of included women

	*n* (14)	*n* (6)	*n* (21)
Maternal age (years) Mean (95% CI)	30.3 (27.4;33.2)	31.0 (27.6; 34.4)	28.9 (26.8; 30.9)
Maternal BMI (kg/m^2^) Mean (95% CI)	23.4 (19.4;27.4)	25.3 (17.7; 33.0)	24.3 (22.2; 26.4)
Nulliparity *n* (%)	10 (77)	5 (83)	15 (71)
Daily caffeine intake *n* (%)	1 (7)	0 (0)	2 (10)
Intensity exercise *n* (%)	0 (0)	0 (0)	0 (0)
Smoking *n* (%)	1 (7)	0 (0)	2 (10)
Fetal gender (male) *n* (%)	8 (57)	4 (67)	11 (52)
AC (*z*‐score) Mean (95% CI) (range)	−2.30 (−2.8; −1.9) (−4.4; −1.0)	−2.46 (−3.53; −1.39) (−4.4; 1.64)	No
UA (*z*‐score)			
Mean (95% CI) (range)	1.34 (0.27; 2.41) (−1.2; 3.1)	2.00 (0.8; 3.3) (−0.2; 3.1)	No
A. umb. (*z*‐score)			
Mean (95% CI) (range)	2. 31 (0.66;3.95) (−1.3;9.1)	3.9 (−0.10; 7.96) (−1.3; 9.1)	No
MCA (*z*‐score)			
Mean (95% CI) (range)	−1.20 (−1.96; −0.44) (−3.6; 1.6)	−2.01 (−3.02; −1.13) (−3.6; −1.3)	No
CPR			
Mean (95% CI) (range)	1.26 (0.92; 1.59) (0.26; 2.11)	0.9 (0.2; 1.6) (0.26;2.11)	No
GA birth (week)			
Mean (95% CI)	35.4 (32.9; 38.0)	31.3 (27.4; 35.3)	40.3 (39.7; 40.8)
Birthweight (gram)	1874 (1458; 2291)		3683 (3495; 3871)
Mean (95% CI)		1191 (619; 1764)	
Birthweight (*z*‐score)	−2.6 (−2.9; −2.2)		0.2 (−0.2; 0.7)
Mean (95% CI)		−3.1 (−3.7; −2.6)	
NICU			
*n* (%)	7 (50)	6 (100)	2 (10)
Perinatal death			
*n* (%)	1 (7)	1 (17)	0 (0)

*Note*: All FGR: Birth weight <2.3 percentile of mean for gestational age and gender. Severe FGR: Birth weight <2.3 percentile of mean for gestational age and gender and delivery before gestational age < 37^+0^. AGA: Birth weight between the 10th and 90th percentile of mean for gestational age and gender.

Abbreviations: AC, Abdominal circumference; CPR, Cerebro‐placental ratio; GA, Gestational age; MCA, Middle cerebral artery flow; UA, Arteria Uterina flow; A. Umb, A umbilicalis flow.

**FIGURE 2:**
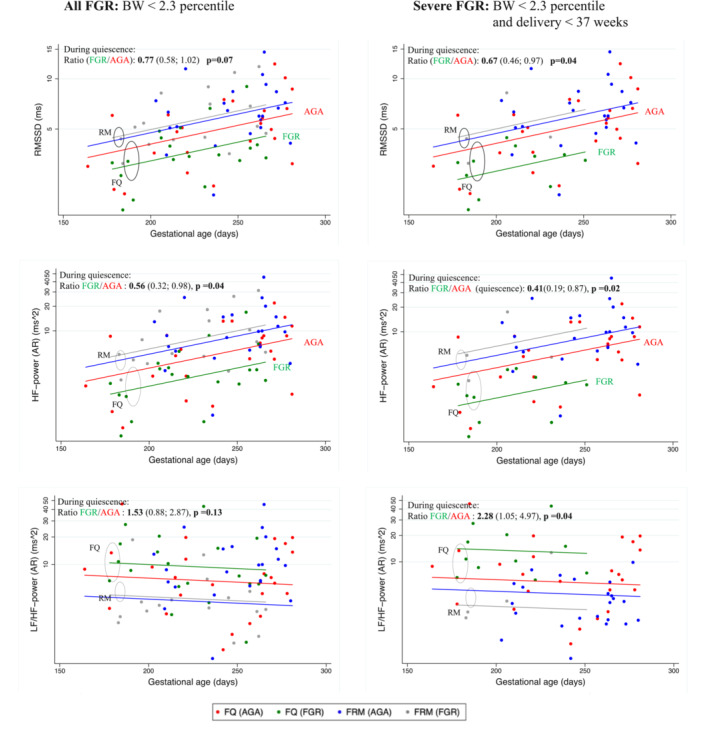
Scatterplot (logscale) and median lines of RMSSD, HF power and LF/HF in two groups of growth‐restricted fetuses (FGR) and average for gestational age fetuses (AGA) in relation to gestational age and fetal movements. Median curves for outcomes as a function of independent variables, gestational age and fetal movements were based on a linear mixed model on the log‐transformed data. FGR and FQ (*n* = 20); Severe FGR and FQ (*n* = 11) AGA and FQ (*n* = 24). FGR and FRM (*n* = 16); Severe FGR and FRM (*n* = 4); AGA and FRM (*n* = 23). BW, Birth weight; FGR, Fetal growth restriction; AGA, average for gestational age; FQ, Fetal quiescence; FRM, Fetal respiratory movements.

FGR fetuses displayed an increase in both time domain and spectral domain parameters with increasing gestational age (Figure 2). Compared to fetal quiescence (FQ), fetal respiratory movements (FRM) were associated with an increase in RMSSD (Ratio: 1.54 [1.33; 1.79]) and HF power (Ratio: 2.88 [2.12; 3.91]) and a decrease in LF/HF (ratio: 0.44 [0.31; 0.63]; Table 2 and Figure 2).

**TABLE 2:** Time domain and spectral domain parameters in fetal growth restriction (FGR); time series obtained during fetal respiratory movements compared to fetal quiescence

	All FGR		Severe FGR	
	Ratio (95% CI) FRM (16)/FQ (20)	*p*‐value	Ratio (95% CI) FRM (4)/FQ (11)	*p*‐value
Mean RR (ms)	1.01 (0.99; 1.03)	0.36	1.01 (0.98; 1.05)	0.40
SDNN (ms)	1.17 (1.00; 1.35)	0.26	1.14 (0.90; 1.45)	0.27
RMSSD (ms)	1.54 (1.33; 1.79)	0.00	1.80 (1.40; 2.30)	0.00
LF‐power (ms^2^)	1.17 (0.84; 1.63)	0.35	1.02 (0.61; 1.69)	0.95
HF‐power (ms^2^)	2.88 (2.12; 3.91)	0.00	4.34 (2.67; 7.07)	0.00
LF/HF‐power	0.44 (0.31; 0.63)	0.00	0.23 (0.15; 0.33)	0.00

*Note*: All FGR: Birth weight <2.3 percentile. Severe FGR: Birth weight <2.3 percentile and delivery before gestational age < 37^+0^.

Abbreviations: 95% CI, 95% confidence interval; BW, birth weight corrected for gestational age and gender (20); FRM, fetal respiratory movements.

Compared to FQ in AGA fetuses, the FQ in FGR fetuses was associated with a trend toward lower RMSSD (Ratio: 0.77 [0.58; 1.02]) and a significantly lower HF power (Ratio 0.56 [0.32; 0.98]). Further, this association was even more pronounced in fetuses with severe FGR (Table 3), and the ratio for RMSSD was significant (Ratio: 0.67 [0.46; 0.97]).

**TABLE 3:** Time domain and spectral domain parameters in growth‐restricted fetuses (FGR) compared to average for gestational age fetuses (AGA), shown for each fetal movement pattern and severity of FGR

	Fetal quiescence (FQ)		Fetal respiratory movements (FRM)		Ratio (FRM/FQ)	
	Ratio (95% CI) FGR /AGA	*p*‐value	Ratio (95% CI) FGR/AGA	*p*‐value	Ratio (95% CI) FGR/AGA	*p*‐value
All FGR: BW <2.3 percentile						
**Recording**	**(*n***_**FGR**_ **= 20) (*n***_**AGA**_ **= 24)**		**(*n***_**FGR**_ **= 16) (*n***_**AGA**_ **= 23)**			
Mean RR	1.02 (0.98; 1.07)	0.34	1.02 (0.97; 1.07)	0.42	0.98 (0.97; 1.02)	0.85
SDNN	0.91 (0.69; 1.18)	0.47	1.04 (0.79;1.38)	0.77	1.14 (0.93; 1.40)	0.2
RMSSD	0.77 (0.58; 1.02)	0.07	1.04 (0.78;1.39)	0.79	1.32 (1.07;1.62)	0.00
LF‐power	0.91 (0.50; 1.64)	0.76	1.11 (0.60; 2.05)	0.75	1.19 (0.75; 1.89)	0.45
HF‐power	0.56 (0.32; 0.98)	0.04	1.16 (0.65;2.06)	0.61	1.98 (1.25; 2.95)	0.00
LF/HF‐power	1.53 (0.88; 2.67)	0.13	1.08 (0.60;1.94)	0.81	0.76 (0.46; 1.24)	0.27
Severe FGR: BW <2.3 percentile and delivery <37 weeks						
**Recordings**	**(*n***_**FGR**_ **= 11) (*n***_**AGA**_ **= 24)**		**(*n***_**FGR**_ **= 4) (*n***_**AGA**_ **= 23)**			
Mean RR	1.01 (0.96;1.06)	0.75	1.01 (0.96; 1.07)	0.68	1.00 (0.97; 1.04)	0.82
SDNN	0.88 (0.61; 1.27)	0.50	0.98 (0.64; 1.52)	0.94	1.12 (0.85; 1.48)	0.43
RMSSD	0.67 (0.46; 0.97)	0.04	1.06 (0.69; 1.63)	0.78	1.59 (1.17; 2.08)	0.00
LF‐power	0.91 (0.40; 2.08)	0.82	0.92 (0.36; 2.38)	0.86	1.02 (0.57; 1.84)	0.95
HF‐power	0.41 (0.19; 0.87)	0.02	1.30 (0.55; 3.03)	0.55	3.04 (1.72; 5.39)	0.00
LF/HF‐power	2.28 (1.05; 4.97)	0.04	0.67 (0.30; 1.52)	0.34	0.31 (0.20; 0.49)	0.00

*Note*: Spectral domain parameters by the autoregressive model.

Abbreviations: 95% CI, 95% conficence interval; AGA, Average for gestational age; BW, Birth weight corrected for gestational age and gender; FGR, Fetal growth restriction.

However, during FRM, FGR fetuses displayed no significant difference in any parameters compared to AGA fetuses, even though a trend toward higher HF power during FRM in the FGR fetuses as compared to the AGA fetuses was seen (Table 3).

The ratios (FRM/FQ) for RMSSD and HF power were higher in FGR fetuses as compared to AGA fetuses, and this association also increased with the severity of FGR (Table 3).

**FIGURE 3:**
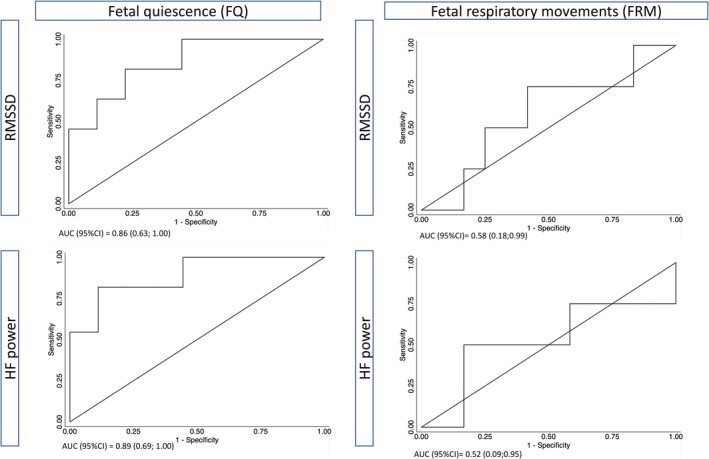
Receiver‐operating characteristics (ROC‐curves) of RMSSD and HF power for prediction of severe FGR (preterm and BW < −22%) within the FGR fetuses, shown for time series obtained during fetal quiescence (FQ) and during fetal respiratory movements (FRM). All FGR and FQ (*n* = 20); Severe FGR and FQ (*n* = 11). All FGR and FRM (*n* = 16); Severe FGR and FRM (*n* = 4). AUC, Area under the receiver operating curve; 95% CI, 95% confidence interval. Non‐parametric bootstrap was applied to adjust AUC confidence intervals for clustering. (correlation) of fetuses.

ROC curves of RMSSD and HF power (AR), obtained during fetal quiescence showed AUC between 0.86 and 0.89 for prediction of severe FGR (BW <2.3 percentile and preterm) within FGR fetuses (Figure 3). This finding reached statistical significance (*p* < 0.05) for both RMSSD and HF power. During fetal respiratory movements, AUC was close to 0.5.

Median curves for outcomes as a function of independent variables, gestational age, and fetal movements were based on a linear mixed model on the log‐transformed data.

## 4 DISCUSSION

### 4.1 Main findings

FGR fetuses demonstrate a respiratory‐dependent increase in RMSSD and HF power and a decrease in LF/HF power of similar magnitude compared to AGA fetuses. However, during fetal quiescence, FGR fetuses display significantly lower RMSSD (only significant in severe FGR) and HF power than AGA fetuses, a reduction which is associated with the severity of the FGR. During fetal quiescence, the diagnostic performance of RMSSD and HF power demonstrated AUC at 0.86 and 0.89, respectively, for predicting severe FGR.

### 4.2 Strengths and limitations

It is a strength that we used an FGR definition based on birthweight <2.3 percentile, which is internationally accepted as an appropriate cut‐off for FGR (Gordijn et al., 2016), a highly standardized method for FHRV assessment, and continuous ultrasound examination for categorization of fetal movements. It is a limitation that three participants received two doses of 12 mg betamethasone administered within a 24‐h interval for fetal lung maturation, as betamethasone may effect FHRV in the first few days after administration (Multon et al., 1997; Noben et al., 2019). This might have affected 4 of the 11 recordings in the group with severe FGR (two were obtained 2 days, and two were obtained 7 days after betamethasone administration). However, our results were unchanged or even more significant (FGR/AGA [quiescence]; Ratio's RMSSD: 0.62, HF power: 0.38) if we exclude these four recordings.

### 4.3 Interpretation and relation to other studies

**FIGURE 4:**
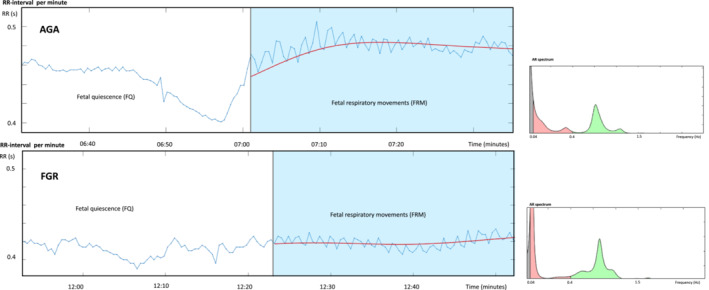
Examples of fetal respiratory sinus arrythmia in an average for gestational age fetus (AGA) (upper) and a growth‐restricted fetus (FGR) (lower). Fetal RR‐intervals (seconds in relation to time (minutes) are shown. The blue‐colored part of the graph represents the RR‐intervals for the AR spectrum.

In this study, FGR fetuses displayed a respiratory‐dependent sinus arrhythmia. This arrhythmia was characterized by increases in parameters known to represent cardiac vagal activity (RMSSD and HF power) during fetal respiratory movements, which was comparable to or even higher than in AGA fetuses. This could also be reflected in the display of RR‐intervals (Figure 4) Most probably, this finding reflects a normally developed and well‐functioning cardiac autonomic nervous system in these FGR fetuses (Arias‐Ortega et al., 2016), all of which (even the most compromised fetuses) also demonstrated long‐lasting periods of respiratory movements at early gestational ages. This is in accordance with a former study, which found higher values of RMSSD and pNN in high‐risk pregnancies (including FGR pregnancies) compared to low‐risk pregnancies, indicating an enhanced maturation at an earlier gestation in the high‐risk pregnancy (Govindan et al., 2007).

During fetal quiescence, however, we found significantly lower RMSSD and HF power in FGR fetuses as compared to AGA fetuses. This correlation could be either a consequence of FGR or a characteristic of FGR fetuses. However, the correlation increased with severity of FGR, which indicates that the correlation between low RMSSD and HF power during fetal quiescence is a consequence of FGR. Furthermore, lower RMSSD and HF power in FGR fetuses is consistent with a suppressed cardiac vagal activity and an increased sympatho‐vagal balance, which implies an activated stress response. One prior study reported a similar trend (Schneider et al., 2006) whereas another study did not (Arias‐Ortega et al., 2016). The discrepancy between the findings in these two studies and those in the present study may be related to differences in the populations. Thus, the birth weight cut‐offs were the 7th and the 10th percentiles (2.3 percentile in this study), resulting in very high fractions of fetuses with normal Doppler flows (our cases had abnormal Doppler flows). Furthermore, our strict definition of fetal quiescence based on continuous ultrasound detection of movements and a stationary heart rate (SHRP) may be decisive. In the postnatal period, most studies compare HRV in AGA neonates with SGA neonates (birth weight below 10th centile), and these results also suggest that SGA is associated with higher sympathetic activity compared to AGA in the postnatal period (Javorka et al., 2017). In early school‐age children, Aye et al. did not find an association between being born SGA and HRV (Aye et al., 2018), whereas Korkalainen et al. found lower SDNN, HF power, VLF power in early school age children born FGR with cerebroplacental ratio (CPR) < 2SD, compared to children born SGA with CPR >= 2SD (Korkalainen et al., 2021). However, GA at delivery was not corrected in these analyses. Studies in neonates and children born SGA points toward the same association we found, suggesting higher sympathetic activity in SGA neonates/children compared to AGA neonates/children.

Severe FGR is associated with hypoxia, which is also reflected in the brain sparring seen in these fetuses. During delivery, an acute hypoxia may develop. This is reflected in a low umbilical artery PH. Van Laar et al. found a significant association between low PH and higher sympathetic measurements of HRV (van Laar et al., 2010). Even though the pathogenesis probably is different in acute hypoxia as compared to the chronic hypoxia seen in FGR, these results also support the theory of an altered FHRV toward higher sympathetic activity in the compromised fetus.

Our finding of a stress response, which can be detected only during fetal quiescence, is also supported by studies in chronically instrumented fetal sheep (Shaw et al., 2018). When lambs were exposed to chronic hypoxia, a lower HF power was found during quiet sleep (HRP I) but not during active sleep (HRP II). This study, however, differed from our study in some important respects. The HF frequency band was set different from ours (0.15–0.4 Hz), which is too low for detection of respiratory‐dependent oscillations in human fetuses and maybe also in fetal sheep. Furthermore, the categorization into quiet sleep and active sleep was based on HRP, which does not consider fetal respiratory movements, as these may occur in all HRP (Pillai & James, 1990). However, they used a time series of 5 min, which probably reveals more LF power than the 64‐second time series used in our study, and in contrast to our findings, they found a lower LF power in the hypoxic group as compared to the normoxic group.

### 4.4 Origin of RSA

From a physiological perspective, our results contribute new knowledge. During fetal quiescence the basic cardiac outflow is probably reflected, and our findings of a suppressed RMSSD and HF power in the FGR fetuses during fetal quiescence but not during fetal respiratory movements therefore suggests a separated regulation of the basic cardiac outflow and the vagally mediated RSA, at least in the fetus. This hypothesis has also been raised by Hayano and Yasuma (2003), who noted that in certain conditions, respiratory vagal modulation of the heart and cardiac vagal tone dissociate. An example is during baroreceptor stimulation, which causes both bradycardia and lower RSA.

In fetuses, regulation of pulmonary ventilation and perfusion is very different from neonates and adults, where the varying vagal efferent output to the heart causing the RSA is a part of optimizing the exchange of oxygen and CO_2_ (Hayano & Yasuma, 2003). In fetuses, the mechanism of RSA is unknown. RSA could be an intrinsic function of the cardiovascular system, which apparently is unaffected by fetal compromise and, thereby, is probably also unaffected by chronic hypoxia or even acidemia.

### 4.5 Clinical perspective

From a clinical perspective our results indicate that NI‐FECG constitutes a potential approach for fetal monitoring. Thus, it might predict adverse neonatal outcome at comparable levels or even better than cCTG evaluation of short‐term variability (STV) (Huhn et al., 2011; Nijhuis et al., 2000) and Doppler flows in the venous duct (Bilardo et al., 2004). This is because the area under the ROC curve is 0.71 for STV and 0.75 for the Doppler flows as compared to 0.86 (RMSSD) and 0.89 (HF) in our study. However, the comparison of these Areas under the ROC curves is hampered by very different populations and very different outcomes in the studies.

This study suggests FHRV as a clinical marker of severity of FGR, which could have high value in the management of women carrying a growth‐restricted fetus, which includes, timing of fetal lung maturation by glucocorticoids, fetal neuroprotection by magnesium sulfate, and ultimately a preterm delivery.

## 5 CONCLUSION

Controlling for fetal movements, especially fetal respiratory movements, is crucial to the FHRV results, also in FGR fetuses. During fetal respiratory movements, FGR fetuses preserve high levels of parameters supposed to represent vagal activity at similar levels as seen in AGA fetuses.

In contrast, during fetal quiescence, FGR fetuses display significantly lower levels of the same parameters as compared to AGA fetuses.

RSA may constitute an intrinsic function of the cardiovascular system, which apparently is unaffected by fetal compromise and, thereby, chronic hypoxia as well, whereas the basic cardiac outflow assessed during fetal quiescence reveal a suppressed cardiac vagal activity and an increased sympatho‐vagal balance in the FGR fetuses.

This study will hopefully serve as a base for future studies evaluating the association between FHRV and FGR. We conclude that FHRV has the potential to be a diagnostic measure for FGR.

However, a larger study, including an assessment of the association between FHRV and abnormal Doppler flows and neonatal outcome, is recommended.

### AUTHOR CONTRIBUTION

Zizzo AR, Uldbjerg N, Kirkegaard I, Petersen OB, Hansen J and Mølgaard H contributed to the conception and design of the project. Zizzo AR did the inclusion of participant, obtained recordings and performed the analyses. Zizzo AR and Mølgaard H did the statistical analyses. All authors contributed to the interpretation of results, writing process and revised the paper critically for important intellectual content. All authors gave final approval of the version to be submitted, agreed to be accountable for all aspects of the work and are designated as authors who qualify for authorship.

### CONFLICTS OF INTEREST

No conflicts of interest.

## ETHICS

The study has been approved by the Danish Data Protection Agency (1‐16‐02‐440‐15) and the Danish National Committee on Health Research Ethics (1‐10‐72‐227‐15).

## REGISTRATION

The study was registered at Clinicaltrials.gov. ID: NCT04288037.
